# Comparison of therapeutic effects between topical 8-oxo-2′-deoxyguanosine and corticosteroid in ocular alkali burn model

**DOI:** 10.1038/s41598-021-86440-7

**Published:** 2021-03-25

**Authors:** Dong Hyun Kim, Sang-Taek Im, Jin Young Yoon, Seunghoon Kim, Mee Kum Kim, Myung-Hee Chung, Chul-Kyu Park

**Affiliations:** 1 Department of Ophthalmology, Gil Medical Center, Gachon University College of Medicine, 1198, Guwol-dong, Namdong-Gu, Incheon, 21565 Korea; 2 Fight Against Angiogenesis Related Blindness (FARB) Laboratory, Seoul National University Hospital, Seoul, Korea; 3 Gachon Medical Research Institute, Gachon University Gil Medical Center, Incheon, Korea; 4 RudaCure Co., Ltd, Incheon, Korea; 5 Department of Ophthalmology, Seoul National University College of Medicine, Seoul, Korea; 6 Neuroscience Research Institute, Gachon University, Incheon, Korea; 7 Gachon Pain Center and Department of Physiology, Gachon University College of Medicine, Incheon, 21999 Korea

**Keywords:** Preclinical research, Eye diseases

## Abstract

We compared the therapeutic effects of topical 8-oxo-2′-deoxyguanosine (8-oxo-dG) and corticosteroid in a murine ocular alkali burn model. (n = 128) The corneal alkali burn model was established by applying 0.1 N sodium hydroxide (NaOH), followed by treatment with 8-oxo-dG, 0.1% fluorometholone (FML), 1% prednisolone acetate (PDE), or phosphate-buffered saline (PBS) twice daily. One week later, the clinical and histological status of the cornea were assessed. Transcript levels of inflammatory cytokines and nicotinamide adenine dinucleotide phosphate (NADPH) oxidase as well as the levels of reactive oxygen species (ROS) and reactive nitrogen species (RNS) in the cornea, were assayed. The 8-oxo-dG and PDE groups showed marked improvements in corneal integrity and clarity when compared with the PBS group (each *p* < 0.01). The numbers of cells stained for neutrophil elastase and F4/80-positive inflammatory cells were significantly decreased, with levels of interleukin(IL)-1β, IL-6, tumor necrosis factor(TNF)-α, and total ROS/RNS amounts markedly reduced in the 8-oxo-dG, FML, and PDE groups (each *p* < 0.05). Levels of NADPH oxidase type 2 and 4 were substantially more repressed in the 8-oxo-dG-treated group than in the PDE-treated group (each *p* < 0.05). Topical 8-oxo-dG showed excellent therapeutic effects that were comparable with those treated with topical PDE in a murine ocular alkali burn model.

## Introduction

Currently, topical corticosteroids are reportedly the most potent anti-inflammatory agents available for treating ocular surface inflammation. Although T cell inhibitors such as cyclosporin A and tacrolimus, are effective against ocular surface inflammation, their anti-inflammatory effects are not as potent as those treated with corticosteroids, especially in acute exacerbation of ocular inflammation^1,2^. However, long-term use of topical corticosteroid is clinically restricted owing to potential risks such as secondary infection, elevated intraocular pressure, and cataract formation^3^. Hence, numerous researchers have investigated to develop safe and potent anti-inflammatory agents as substitutes for topical corticosteroids.

8-oxo-2′-deoxyguanosine (8-oxo-dG) is an oxidized derivative of deoxyguanosine that is released through a chemical reaction in DNA when the guanine base is damaged^4^. Notably, 8-oxo-dG increases in both mitochondrial and nuclear DNA with aging, and contributes to carcinogenesis by modulating gene expression or inducing mutations^5,6^. Moreover, 8-oxo-dG has been established as a biomarker for oxidative damage in diverse disease conditions including atherosclerosis, diabetes mellitus, and various cancers^7–10^. Interestingly, previous studies have demonstrated that exogenous 8-oxo-dG did not affect DNA synthesis, and revealed potent anti-inflammatory and anti-oxidative effects in several disease models via Rac1 inhibition^4,11,12^. Accordingly, 8-oxo-dG could be used as both an oxidative stress biomarker and a therapeutic agent for inflammatory or oxidative stress-related diseases.

Ocular chemical burns are potentially blinding conditions that require immediate and intensive intervention to minimize severe complications^13,14^. Two main strategies for managing ocular alkali burn include promoting epithelial healing and suppressing inflammation during the acute phase^13,14^. Ocular chemical burns reportedly increase the levels of several pro-inflammatory cytokines such as interleukin (IL)-1, IL-6, and matrix metalloproteinase (MMP)-9, which induce immune cell infiltration into tissues and disrupt the delicate balance between pro-angiogenic and anti-angiogenic factors^15–17^. Therefore, several studies have used the ocular alkali burn models to investigate acute ocular surface inflammation^18–21^. Furthermore, this model is widely used to assess the efficacy of anti-inflammatory molecules for the development of novel anti-inflammatory agents^13,22^.

Recently, we have reported that topical 8-oxo-dG dose-dependently promotes healing of the corneal epithelium and suppresses inflammation in ethanol-induced ocular injury model^23^. In the present study, we compared the therapeutic effects of topical 8-oxo-dG and corticosteroids in a murine ocular alkali burn model to assess the anti-inflammatory efficacy of 8-oxo-dG as a follow-up study. Herein, 1% prednisolone, known to be the most powerful anti-inflammatory agent in commercially available eyedrops, was included as a corticosteroid agent.

## Methods

### Animals and experimental design

All experimental animals were handled according to the Guide for the Care and Use of Laboratory Animals of the National Institutes of Health, and experimental protocols were approved by the Institutional Animal Care and Use Committee of Lee Gil Ya Cancer and Diabetes Institute (LCDI-2017-0046). Animals were treated in strict accordance with the Association for Research in Vision and Ophthalmology Statement for the Use of Animals in Ophthalmic and Vision Research. Principles outlined in the ARRIVE guideline were considered when planning the experiments. (Supplementary file 1, ARRIVE guideline checklist).

Eight-week-old female BALB/c mice were used in these experiments (Orient Bio Inc, Seongnam, Korea). All mice were maintained at the animal facility of Lee Gil Ya Cancer and Diabetes Institute under a specific pathogen-free environment with free access to water and food. Anesthesia was induced with an intramuscular injection of tiletamine and zolazepam (30 mg/kg, Zoletil 50, Virbac, Carros, France) and xylazine hydrochloride (5 mg/kg). In the right eye of each mouse (n = 128), an ocular alkali burn was created by applying a 3 mm piece of filter paper soaked in 0.1 N NaOH to the corneal center for 15 s, with the cornea then sufficiently rinsed with phosphate-buffered saline (PBS) for 60 s.

The mice were treated with the following four ophthalmic solutions: 50 and 10 mg/mL 8-oxo-dG, fluorometholone (FML) 0.1% (1 mg/mL FML, Fluvin, Taejoon Pharmaceutical, Seoul, Korea), prednisolone acetate (PDE) ophthalmic suspension 1% (10 mg/mL prednisolone acetate, Pred Forte, Allergan, Inc., Irvine, CA, USA). 8-oxo-dG was dissolved in PBS. After establishing the alkali burn model, 10 μL of each topical agent was instilled into the right eye twice daily for 1 week. The same volume of PBS was instilled twice daily into the eyes of the control group mice for 7 days. Topical treatments were initiated 16–24 h after establishing the alkali burn model. The naïve, PBS, 8-oxo-dG (10 mg/mL), PDE, and FML groups each comprised 23 animals, whereas the 8-oxo-dG (50 mg/mL) group comprised 13 animals.

### Clinical examinations

Corneal clarity, epithelial integrity, and neovascularization were clinically evaluated by a corneal specialist (DH Kim), using a handheld slit-lamp biomicroscope (SL-17, Kowa, Tokyo, Japan) 7 days after the chemical injury. (n = 128) Corneal epithelial integrity was evaluated by administering 1 drop of 3% Lissamine Green B (Sigma-Aldrich) to the inferior lateral conjunctival sac, with the the ocular surface then photographed using a Dino-Lite digital camera (Dino-Lite Pro, AnMo Electronics Corp, Hsinchu, Taiwan). Corneal epithelial integrity was scored based on the following scale of 0 to 4 according to the intensity of the corneal epithelial defect: 0 = no epithelial defect, 1 = less than 25% epithelial defect, 2 = 25 to 50% epithelial defect, 3 = 50 to 75% epithelial defect, 4 = more than 75% epithelial defect^18^.

Corneal clarity was scored using the following scale from 0 to 4: 0, no opacity, completely clear cornea; 1 = slightly hazy, iris and lens visible; 2 = moderately opaque, iris and lens still detectable; 3 = severely opaque, iris and lens hardly visible; and 4 = completely opaque, with no view of the iris and lens^19^. Corneal neovascularization was graded on a scale between 0 and 3 with increments of 0.5, using a grid system for each quadrant based on the centripetal extent of the neovascular branches of the limbus. Scores for each quadrant were combined to obtain the neovascularization score (ranging from 0 to 12)^20^.

### Histological_examinations_

After 1 week of treatment, the eyeballs were enucleated, fixed in 10% formalin, and embedded in paraffin. The tissues were cut into 4 µm-thick sections and stained with hematoxylin and eosin (H&E). Furthermore, immunohistochemistry (IHC) staining was performed using anti-neutrophil elastase (marker of neutrophils, Abcam, Cambridge, UK) and anti-F4/80 (a marker of macrophage, Abcam, Cambridge, UK). For IHC, the sections were deparaffinized, rehydrated, and boiled in 10 mM citrate buffer (pH 6) or 1 mM ethylenediaminetetraacetic acid (EDTA) buffer (pH 8) for antibody retrieval. (n = 72).

Next, the sections were incubated with a peroxidase blocking solution (Agilent, Santa Clara, CA, USA) for 10 min. After washing to remove the PBS, the sections were blocked with blocking buffer (Protein Block, Serum-Free, Dako, Denmark) for 10 min at room temperature, followed by overnight incubation in the primary antibody at 4 °C. Then, the sections were incubated with a secondary antibody (REAL EnVision secondary antibody, Dako, Denmark) for 15 min and treated with 3,3′-diaminobenzinide (DAB).

Finally, each section was counterstained with hematoxylin and mounted with Canada balsam. The slides were observed and photographed using a light microscope (BX51, Olympus, Japan). H&E-stained tissue images were quantified to determine the corneal stromal thickness using the Image J software [version 1.44; National Institutes of Health (NIH), Bethesda, MD, USA]. Neutrophil elastase and F4/80-positive cells at 400 × magnification, were counted in four different sections by two independent examiners (DH Kim and JY Yoon), and the average cell count was calculated.

### Total ROS/RNS assay

Total ROS/RNS levels in the excised cornea were measured using OxiSelec ROS/RNS assay kit (Cell Biolabs., Inc., San Diego, CA, USA). (n = 20) The cornea was harvested and immediately homogenized in PBS, centrifuged at 10,000 × g for 5 min, and the supernatant was collected. Then, 50 μL of each supernatant was analyzed using an ROS/RNS assay kit according to the manufacturer’s instructions. The method used was based on the reaction of the fluorogenic probe 2′,7′-dichlorodihydrofluorescin (DCFH)-DiOxyQ. In the cytosol, the probe is deacetylated to the non-fluorescent DCFH, which reacts with ROS and RNS (predominantly H_2_O_2_, ROO·, NO, and ONOO–) to form the fluorescent product 2′,7′-dichlorodihydrofluorescein (DCF). The DCF fluorescence intensity (excitation and emission wavelengths (λex and λem), 480 and 530 nm, respectively) is proportional to the amount of ROS/RNS in the biological sample, which was calculated using a calibration curve based on the standard solution of DCF in PBS. The measurements were performed using a Wallac 1420 Victor 3 (Perkin Elmer, USA) microplate reader. The total ROS/RNS assay was performed 3 days after treatment.

### Real-time reverse transcription-polymerase chain reaction (RT-PCR)

For the reverse transcription-polymerase chain reaction (RT-PCR) analysis of nicotinamide adenine dinucleotide phosphate (NADPH) oxidases (NADPH oxidase type 2 and 4 [Nox2 and Nox4]) and the inflammatory cytokines (IL-1β, IL-6, and TNF-α), the mouse eyeballs were harvested 3 days (n = 16) and 1 week (n = 20) after treatment, respectively. The ocular surface including the cornea and conjunctiva was extracted, lysed in RNA isolation reagent (RNA Bee, Tel-Test, Friendswood, TX, USA), and homogenized using a probe sonicator (VCX 130, Sonics and Materials Inc., Newtown, CT, USA).

Total RNA was extracted using RNeasy Mini kit (Qiagen, Valencia, CA, USA) and quantified using the NanoDrop spectrophotometer. Equal amounts of RNA from each sample were used to synthesize double-stranded cDNA using RT (High Capacity RNA-to-cDNA kit; Applied Biosystems, USA). Real-time RT-PCR (Bio-Rad Laboratories, Roche, Applied Biosystems, USA) was used to analyze the cDNA for the following cytokines: IL-1β (Taqman Gene Expression Assays ID, Mm00434228_m1), IL-6 (Mm00446190_m1), TNF-α (Mm00443260_g1), Nox2 (Mm01287743_m1), and Nox4 (Mm00479246_m1).

18S rRNA (Taqman gene expression assay kit ID, Mn03928990_g1) was used to normalize gene expression. For the PCR probe sets, Taqman gene expression assay kits were purchased from Applied Biosystems. The assays were performed using dual technical replicates of each sample.

### Safety evaluation according to repeated administration of 8-oxo-dG

Six BALB/c female mice were divided into two groups (8-oxo-dG and control) with three mice each and 10 mg/mL of 8-oxo-dG solutions was applied to the right eye at 2 h intervals for 3 days (total of 24 times). The control group was left untreated. Mice in both groups were weighed daily for 3 days and examined for clinical and histological changes.

### Statistical analysis

All measurements between the groups were compared using a one-way analysis of variance (ANOVA, GraphPad Prism, Inc., La Jolla, CA, USA), and are presented as means ± standard error of the mean (SEM). Dunn’s multiple comparisons test was used for additional subgroup analysis and differences were considered statistically significant at *p* < 0.05.

## Results

### 8-oxo-dG and corticosteroid comparably improved clinical parameters

**Figure 1:**
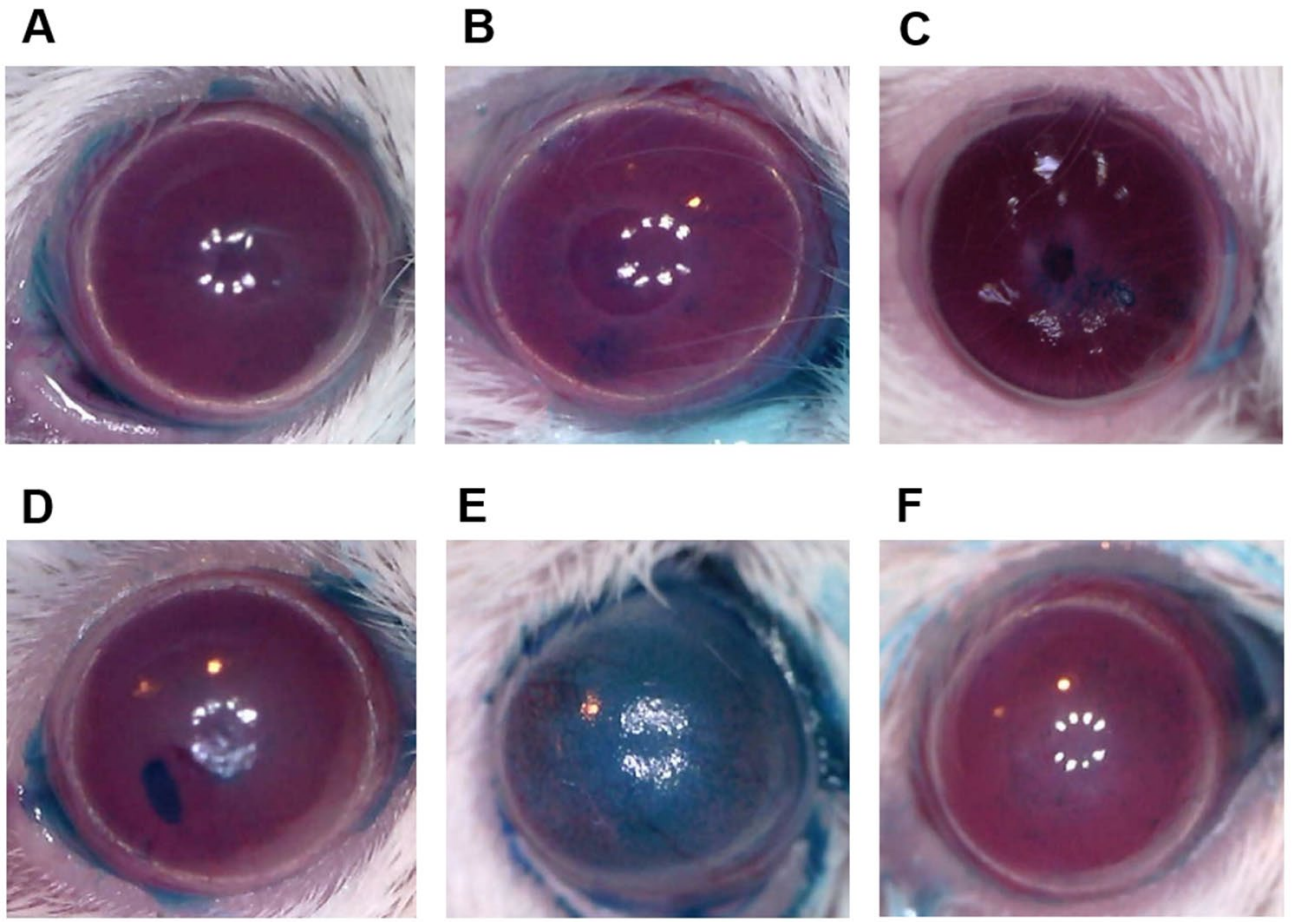
Clinical images of mouse corneas 1 week after treatment of ocular alkali burn model with topical 8-oxo-2′-deoxyguanosine (8-oxo-dG) or corticosteroid. 8-oxo-dG (**A**) 50 mg/mL and (**B**) 10 mg/mL, (**C**) fluorometholone (FML) acetate 0.1% (1 mg/mL), (**D**) prednisolone acetate (PDE) 1% (10 mg/mL), (**E**) PBS, and (**F**) naïve control. *PBS* phosphate-buffered saline.

On day 7, 8-oxo-dG(50 and 10 mg/mL), FML, and PDE groups showed a greater reduction in corneal opacity and epithelial defect 7 than the PBS group (Fig. 1A–E). The PBS group presented prominent epithelial defects and severe stromal haze (Fig. 1E). The 8-oxo-dG group revealed almost complete recovery of the corneal epithelial defect (Fig. 1A and B). The FML group showed a greater stromal haze than the 8-oxo-dG and PDE groups (Fig. 1A–D). Moreover, the 8-oxo-dG treated groups showed an appearance similar to normal cornea of the naïve control group treated with PBS. (Fig. 1A, B, F).

**Figure 2:**
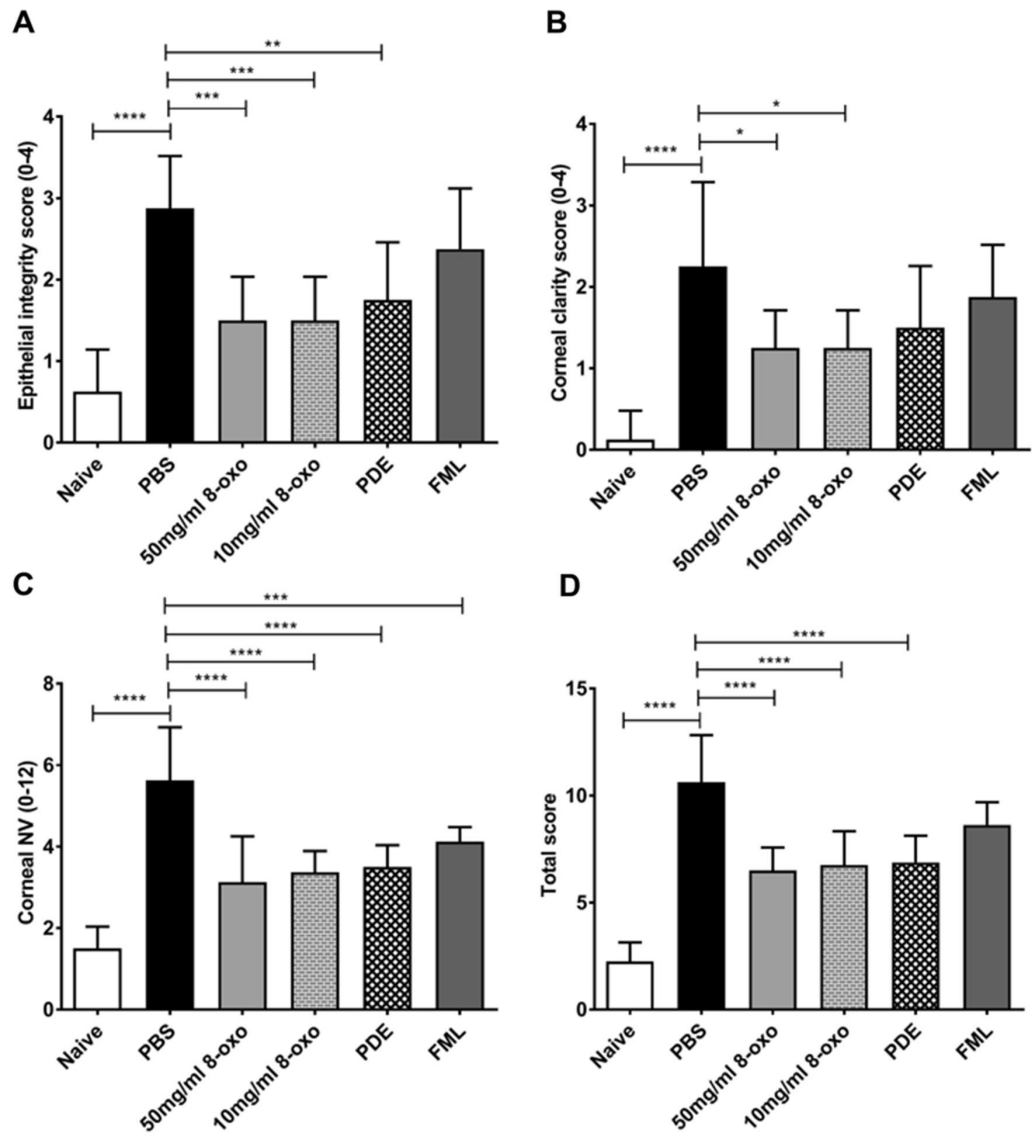
Clinical scores 1 week after treatment of ocular alkali burn model with topical 8-oxo-2′-deoxyguanosine (8-oxo-dG) or corticosteroid. (**A**) Epithelial integrity score, (**B**) corneal clarity score, (**C**) corneal neovascularization score, and (**D**) total score. Data are means ± standard error of the mean (SEM). * p < 0.05, **p < 0.01, ***p < 0.001, and ****p < 0.0001. 8-oxo: 8-oxo-dG. *PBS* phosphate-buffered saline, *PDE* prednisolone acetate 1% (10 mg/mL), *FML* fluorometholone 0.1% (1 mg/mL), *NV* neovascularization.

For the 8-oxo-dG and PDE groups, the corneal epithelial integrity scores were better than that of the PBS group (*p* < 0.001 and *p* < 0.01, 8-oxo-dG and PDE, respectively; Fig. 2A). Furthermore, the 8-oxo-dG group showed better corneal clarity compared to the PBS group (each *p* < 0.05, Fig. 2B). The corneal neovascularization scores of the 8-oxo-dG, FML, and PDE groups were better than that of the PBS group (*p* < 0.0001, 50 and 10 mg/mL 8-oxo-dG and PDE; *p* < 0.001, FML; Fig. 2C). The total clinical score of the 8-oxo-dG, FML, and PDE groups was better than that of the PBS group (each *p* < 0.0001, 50 and 10 mg/mL 8-oxo-dG and PDE; Fig. 2D).

### 8-oxo-dG histologically attenuated inflammation similar to corticosteroids

**Figure 3:**
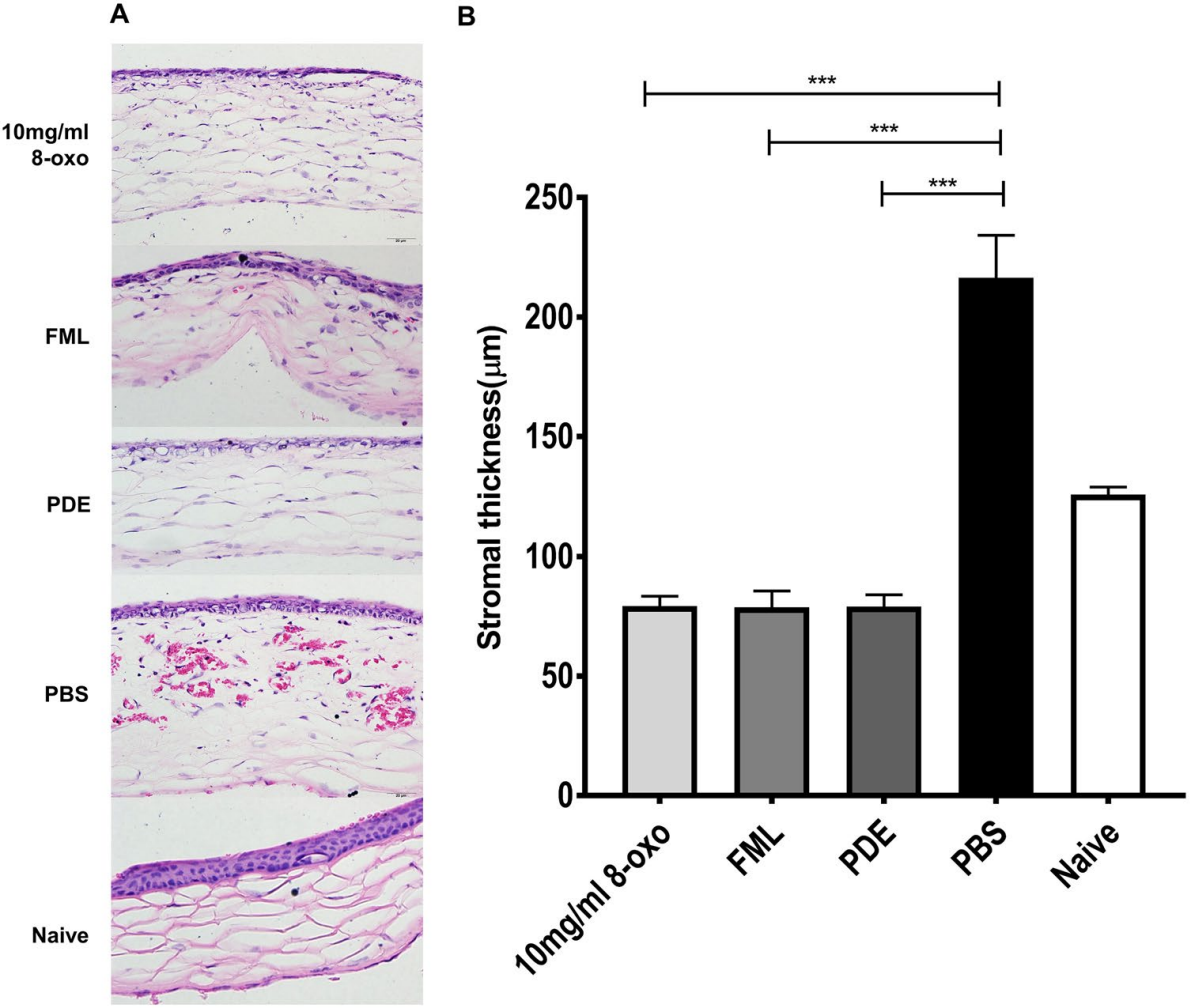
Histological analysis using Image J software [version 1.44; National Institutes of Health (NIH), Bethesda, MD, USA] of the cornea 1 week after treatment of ocular alkali burn model with topical 8-oxo-2′-deoxyguanosine (8-oxo-dG) or corticosteroid. (**A**) Representative images of hematoxylin and eosin (H&E) staining of the central cornea. (**B**) Comparison of stromal thickness of the central cornea. Original magnification, × 400; data are means ± standard error of the mean (SEM). ***p < 0.001. 8-oxo: 8-oxo-dG. *FML* fluorometholone 0.1% (1 mg/mL), *PDE* prednisolone acetate 1% (10 mg/mL), *PBS* phosphate-buffered saline.

H&E staining revealed that the edema in the corneal stroma was more significantly reduced in the 8-oxo-dG (10 mg/mL) and corticosteroid groups than in the PBS group (Fig. 3A and B). The mean stromal thicknesses of the central cornea were 79.3 ± 4.1(mean ± standard error), 78.8 ± 6.8, 79.0 ± 4.9, 216.4 ±, and 125.7 ± 3.2 in the 8-oxo-dG, FML, PDE, PBS, and naïve groups, respectively (*p* < 0.001, Fig. 3B).

**Figure 4:**
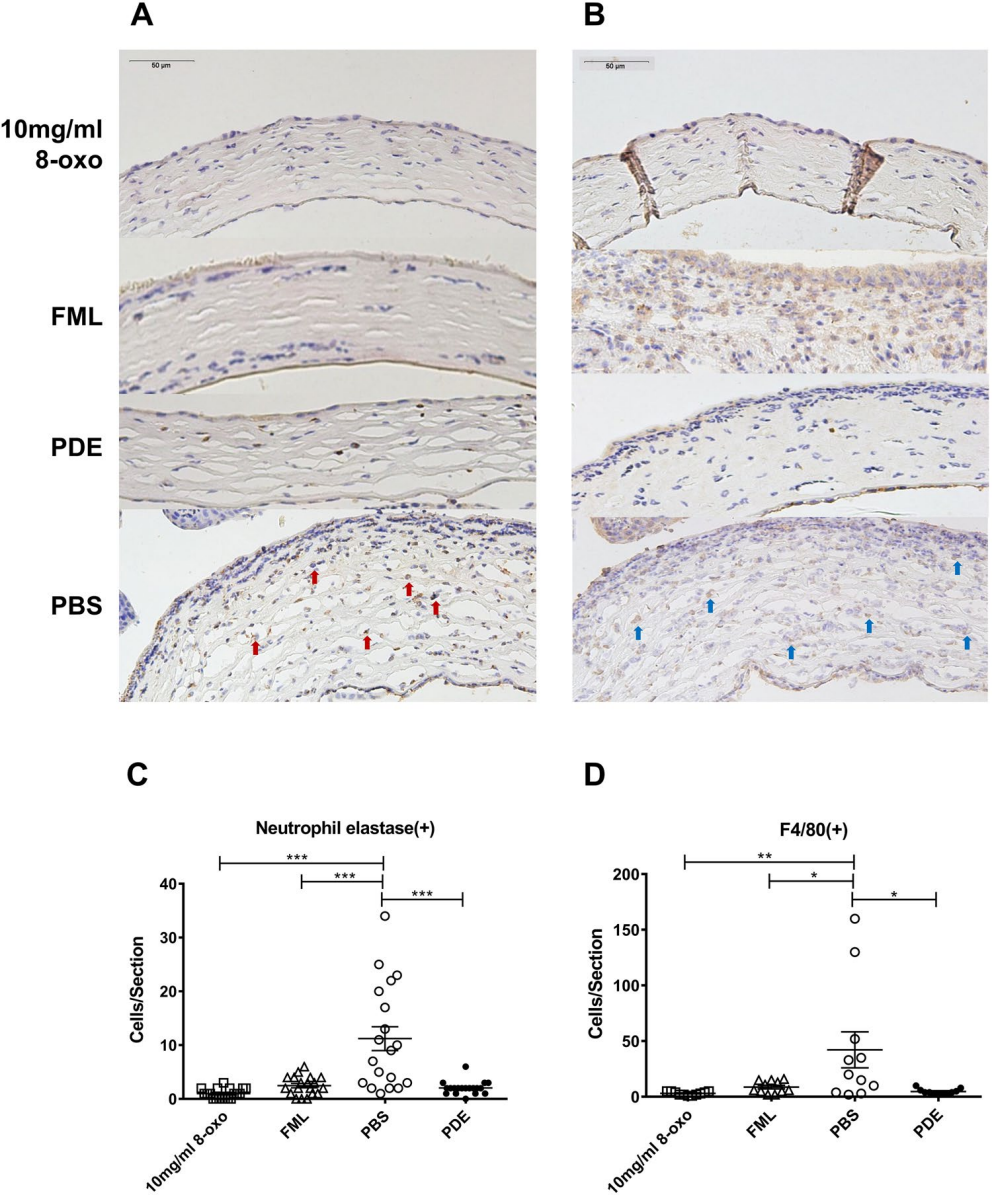
Immunohistological analysis of cornea 1 week after treatment of ocular alkali burn model with topical 8-oxo-2′-deoxyguanosine (8-oxo-dG) or corticosteroid. Representative images of central cornea stained with (**A**) anti-neutrophil elastase and (**B**) anti F4/80 in 8-oxo-dG (10 mg/mL), FML, PDE, and PBS groups. Red arrow: neutrophil elastase positive cells, Blue arrow: F4/80-positive cells. (**C**, **D**) Comparison of neutrophil elastase and F4/80-positive cells in the central cornea. Data are means ± standard error of the mean (SEM). *p < 0.05, **p < 0.01, and ***p < 0.001. 8-oxo: 8-oxo-dG. *FML* fluorometholone 0.1% (1 mg/mL), *PDE* prednisolone acetate 1% (10 mg/mL), *PBS* phosphate-buffered saline.

Furthermore, inflammatory cell infiltration into the corneal stroma was markedly reduced in the 8-oxo-dG and corticosteroid groups compared to in the PBS group (Fig. 3A). The 8-oxo-dG and PDE groups displayed histological findings similar to those of the naïve group, except for the thinning of the epithelial cell layer. The IHC staining showed that there were significantly fewer neutrophil elastase and F4/80-positive cells in the 8-oxo-dG, FML, and PDE groups than in the PBS group (each *p* < 0.001, Fig. 4A and C). In addition, the number of F4/80-positive cells was significantly lower in the 8-oxo-dG, FML, and PDE groups than that in the PBS group (each *p* < 0.05, Fig. 4B and D).

### 8-oxo-dG modulated inflammatory cytokine and oxidative stress-related molecules appeared to be better than corticosteroids

**Figure 5:**
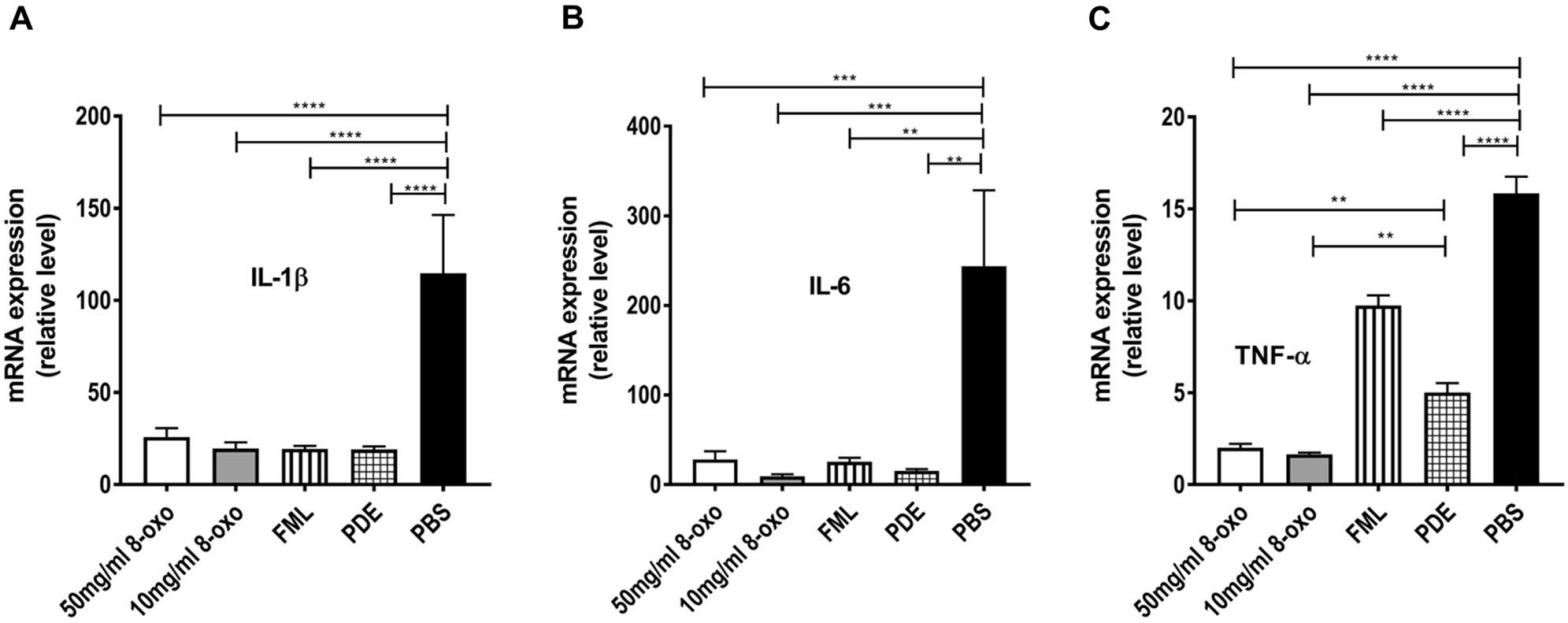
Real-time reverse transcription-polymerase chain reaction (PCR) assay of corneal specimens 1 week after treatment of ocular alkali burn model with topical 8-oxo-2′-deoxyguanosine (8-oxo-dG) or corticosteroid. Relative mRNA expression levels of IL-1β (**A**), TNF-α (**B**), and IL-6 (**C**). Data are presented as means ± standard error of the mean (SEM). **p < 0.01. 8-oxo: 8-oxo-dG. *FML* fluorometholone 0.1% (1 mg/mL), *PDE* prednisolone acetate 1% (10 mg/mL), *PBS* phosphate-buffered saline, *IL* interleukin, *TNF* tumor necrosis factor.

In the excised cornea, transcript levels of inflammatory cytokines (IL-1β, IL-6, and TNF-α) were more markedly reduced in the 8-oxo-dG, FML, and PDE groups than in the PBS group 3 days after treatment (IL-1β, *p* < 0.0001; IL-6, *p* < 0.01; and TNF-α, *p* < 0.0001; Fig. 5A–C). Interestingly, expression levels of TNF-α in the 8-oxo-dG groups (50 and 10 mg/mL) were significantly lower than those in the PDE group (*p* < 0.01, Fig. 5C).

**Figure 6:**
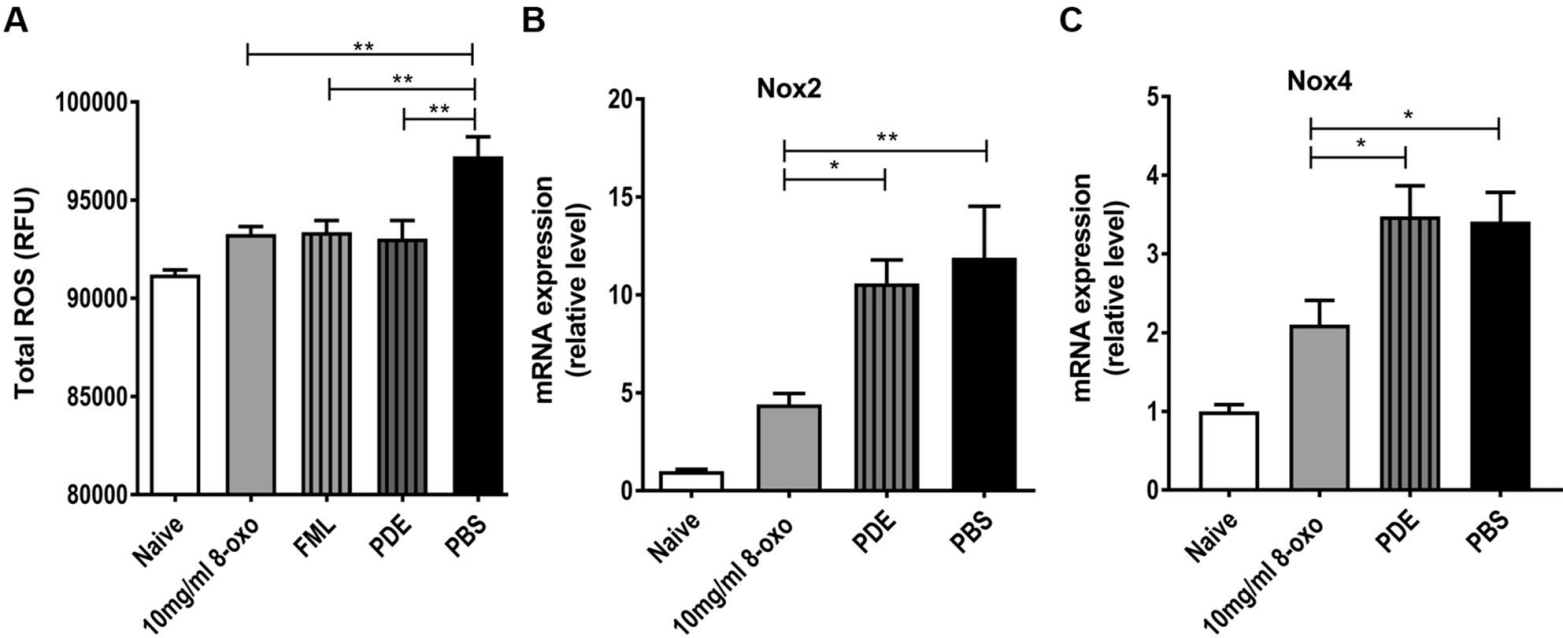
Reactive oxygen species (ROS) and reactive nitrogen species (RNS) assays of corneal specimens 1 week after treatment with topical 8-oxo-dGor corticosteroid in ocular alkali burn models. (**A**) Real-time reverse transcription PCR assays of corneal specimens after treatment of ethanol-induced chemical injury model with 8-oxo-2′-deoxyguanosine (8-oxo-dG). Relative mRNA expression levels of (**B**) Nox2 and (**C**) Nox4. Data are means ± standard error of the mean (SEM). *p < 0.05 and **p < 0.01. RFU: relative fluorescence units. 8-oxo: 8-oxo-dG. *FML* fluorometholone 0.1% (1 mg/mL), *PDE* prednisolone acetate 1% (10 mg/mL), *PBS* phosphate buffered saline, *Nox2* nicotinamide adenine dinucleotide phosphate (NADPH) type 2 oxidase, *Nox4* nicotinamide adenine dinucleotide phosphate (NADPH) type 4 oxidase.

In the 8-oxo-dG (10 mg/mL), FML, and PDE groups, the total ROS/RNS levels in the cornea were significantly lower than those in the PBS group (*p* < 0.01, Fig. 6A). Moreover, the transcript levels of Nox2 and Nox4 were significantly lower in the 8-oxo-dG (10 mg/mL) group than in the PDE and PBS groups (each *p* < 0.05, Fig. 6B and C).

### Short-term repeated administration of topical 8-oxo-dG did not cause adverse clinical and histological changes

**Figure 7:**
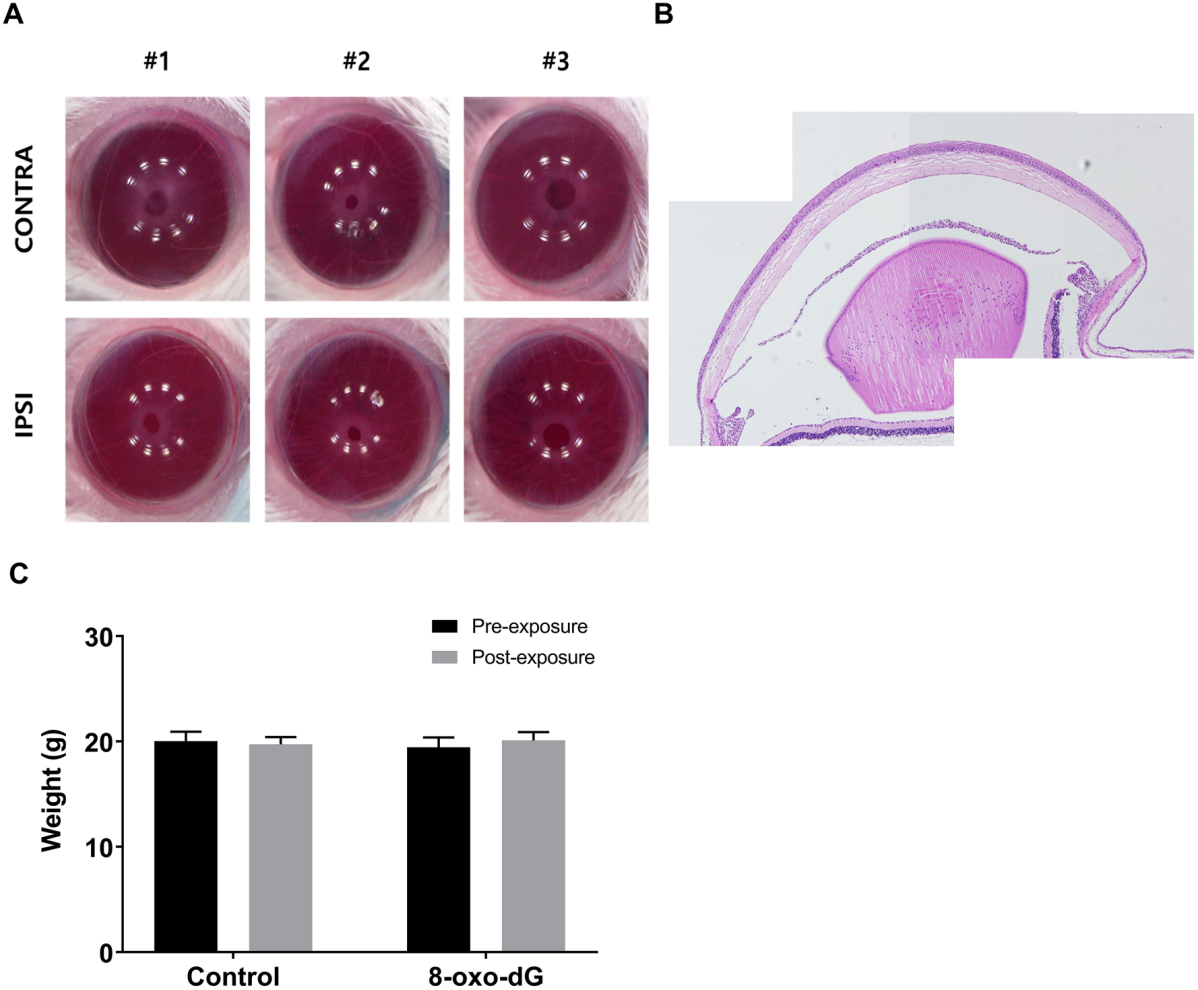
Biological changes observed after repeated administration of topical 8-oxo-2′-deoxyguanosine (8-oxo-dG) for 3 days (total 24 times). (**A**) Clinical images of cornea after repeated exposure. (**B**) Histologic findings of the whole eyeball after repeated exposure of 8-oxo-dG. (**C**) Comparisons of mouse body weight between pre-exposure and post-exposure. *IPSI* application of 8-oxo-dG, *CONTRA* no application.

The 8-oxo-dG group showed similar clinical findings to those observed in the control group without any obvious abnormalities (Fig. 7A). H&E staining showed no abnormal findings in the 8-oxo-dG group (Fig. 7B), with no significant differences between the pre- and post-exposure body weight of both groups (*p* > 0.05, Fig. 7C).

## Discussion

In the present study, topical 8-oxo-dG demonstrated excellent therapeutic effects that were comparable with corticosteroids (especially with PDE) in an experimental ocular alkali burn model. Similar with therapeutic actions of corticosteroids, 8-oxo-dG effectively reduced the severe corneal damage induced by alkali injury including epithelial defect, corneal opacity, and neovascularization. Moreover, 50 and 10 mg/mL 8-oxo-dG showed similar therapeutic effects. In addition, stromal edema, inflammatory cell infiltration, and expression of inflammatory cytokines and oxidative stress-related molecules were significantly decreased after treatment with 8-oxo-dG, and these effects were comparable with those of the corticosteroid. Repeated administration of topical 8-oxo-dG showed no remarkable effects on the cornea and did not induce significant weight changes.

DNA damage induced by ROS readily transforms guanine into 8-oxo-7,8-dihydroguanine (8-oxo-Gua), which is the most abundant oxidative DNA adduct^24^. In DNA, 8-oxo-Gua can have noxious effects owing to the induction of transversion mutation^25^. Fortunately, cells possess relevant repair systems that protect against damage by 8-oxo-Gua. For instance, 8-oxo-Gua in DNA is removed by processes that involve base excision repair enzymes, including 8-oxo-guanine glycosylase, or by a nucleotide excision repair mechanism. Consequently, 8-oxo-dG, a nucleoside of 8-oxo-Gua, is generated from damaged oligomers, including 8-oxo-Gua, or cytoplasmic oxidized nucleotides, such as 8-hydroxy-dGTP^26,27^. Reportedly, 8-oxo-dG is membrane permeable and, thus, typically detected in the urine or sera of patients. Therefore, elevated levels of 8-oxo-dG are generally recognized as a biomarker for oxidative damage in various disease models, including atherosclerosis and diabetes mellitus^7,28–30^. Previous studies have demonstrated that 8-oxo-dG suppresses the activation of neutrophils and macrophages by inhibiting Rac1^11,31,32^. Rac1, a small G protein is involved in modulating the cytoskeleton and signaling pathways in cellular responses^33^. Rac1-linked functions of neutrophils and macrophages include phagocytosis, chemotaxis, inflammatory cytokine release, and ROS production through NADPH oxidase activation^34^. Furthermore, Rac1 appears to regulate the mitogen-activated protein kinase (MAPK) system, extracellular signal-regulated kinase (ERK), c-Jun N-terminal kinase (JNK), Janus kinase (JAK)/signal transducer and activator of transcription (STAT), phosphoinositide 3-kinase (PI3K)/Akt, nuclear factor (NF)-κB pathway, and mechanistic target of rapamycin (mTOR) complex^33^. The molecular mechanism of inflammation is markedly complex, with the entire inflammatory response process mediated by several modulators that are involved in the expression of pro-inflammatory molecules. Therefore, substances that can regulate various inflammatory pathways, for instance, 8-oxo-dG used in the current study, may effectively block inflammation and reduce the associated sequelae.

**Figure 8:**
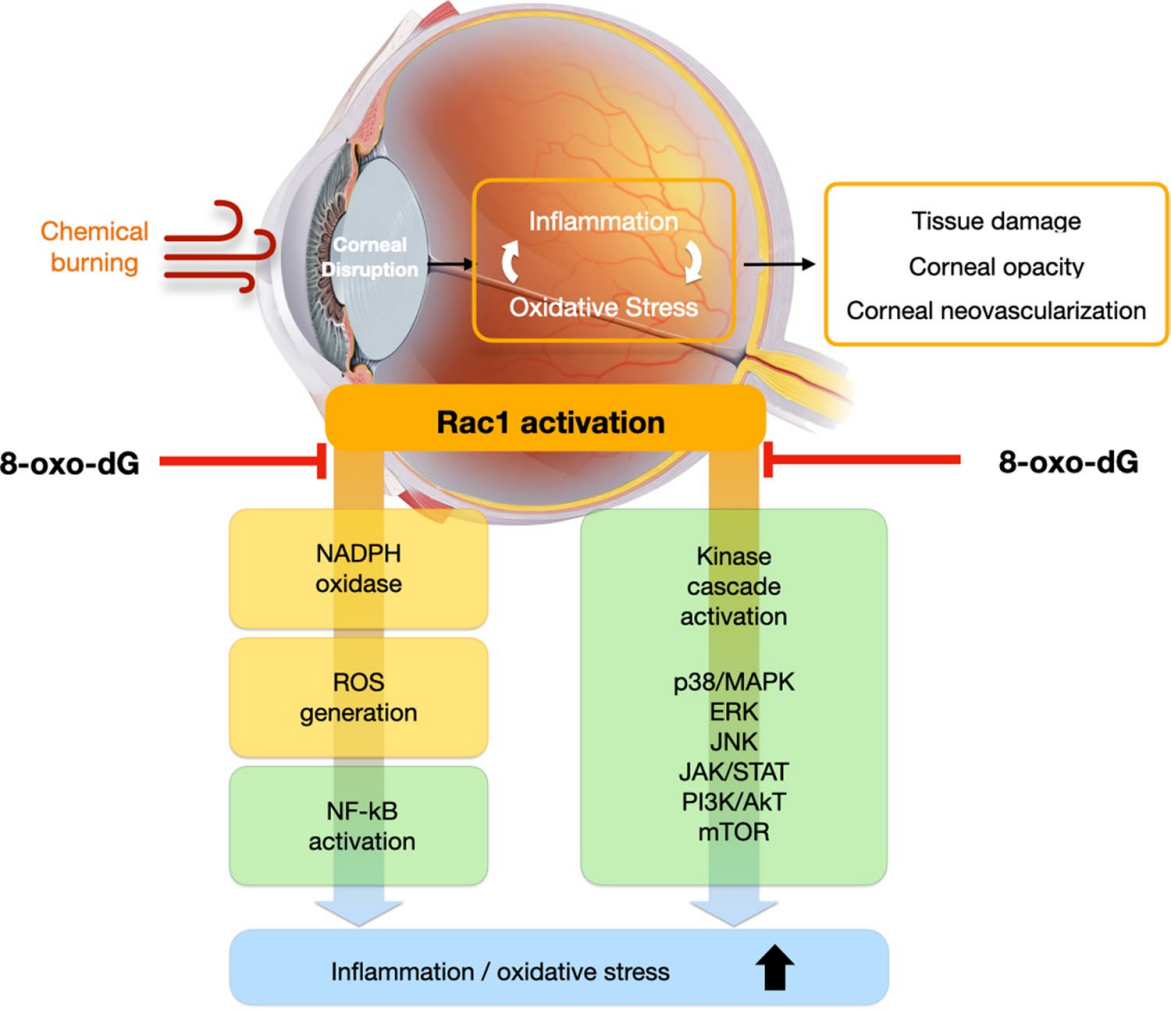
Putative anti-inflammatory mechanism of 8-oxo-2′-deoxyguanosine.

When the ocular surface is injured in the alkali burn model, corneal epithelial cells immediately release numerous inflammatory mediators, such as IL-1, IL-6, TNF-α, RANTES, and ROS. These inflammatory mediators recruit innate immune cells (such as neutrophils and macrophages) to the injured cornea and activate keratocytes that produce chemokines to recruit large amounts of inflammatory cells, which further amplifies pro-inflammatory signals, and ultimately causes irreversible corneal opacity^21,35–37^. Therefore, blocking the early inflammatory process of the corneal epithelial and innate immune cells is critical for effectively treating ocular alkali burn. Corticosteroids are well known to attenuate the innate immune response by inhibiting the activation of antigen-presenting cells and reducing the secretion of pro-inflammatory cytokines. Additionally, corticosteroids reduce the activation of neutrophils and B cells, and modulate T cell polarization and apoptosis^38^. Early treatment with a topical steroid can effectively control acute inflammation and reduce critical complications at the ocular surface by inhibiting various inflammatory cascades. Notably, 8-oxo-dG is thought to demonstrate comparable therapeutic effects with those of PDE by blocking various inflammatory cascades mediated by Rac1 inhibition (Fig. 8)^31–34^.

In the ocular alkali burn model, clinical and histological improvements induced by 8-oxo-dG were comparable with those of PDE, which is the most potent corticosteroid eyedrop commercially available. FML is less potent than PDE, and accordingly, showed a slower and weaker recovery of epithelial integrity and increased stromal haze with an apparent dose–response effect. Interestingly, 50 and 10 mg/mL 8-oxo-dG showed similar improvements. In our previous study, a distinct dose–response relationship was observed at 5 and 10 mg/mL 8-oxo-dG^23^. The maximum effective dose of 8-oxo-dG eye drops is estimated at approximately 10 mg/mL, as 8-oxo-dG would fail to completely dissolve in PBS at higher concentrations and likely form a suspension. The actual concentration of the 8-oxo-dG eyedrops may not be increased because the solute was probably incompletely dissolved at a concentration of 50 mg/mL. IHC staining results revealed that 10 mg/mL 8-oxo-dG and PDE effectively blocked the recruitment of neutrophils and macrophages to the damaged cornea, which is a well-established mechanism of action^23^. IL-1β and IL-6 levels in the 8-oxo-dG, FML, and PDE groups were similarly repressed more than those of the PBS group. TNF-α levels were considerably lower in the 8-oxo-dG group than those in the PDE group. Nox2 and Nox4 activate the NF-κB pathway and stimulate the release of vascular endothelial growth factor (VEGF) and MMPs^37^. The inflammatory molecules, VEGF, and MMPs can cause pathogenic corneal neovascularization and permanent ocular surface damage^37^. In the present study, the levels of Nox2 and Nox4 were more markedly repressed in the 8-oxo-dG group than in the PDE group. This observation indicates that 8-oxo-dG may have more potent anti-inflammatory and anti-oxidative effects than those of PDE. Currently, corticosteroids are widely used to treat ocular inflammation, including those associated with chemical burns, dry eye disease, uveitis, allergic conjunctivitis, and macular edema. Our study also showed that short-term repeated administration of 8-oxo-dG did not cause any noticeable adverse reactions. Therefore, we believe that 8-oxo-dG may be a good alternative to corticosteroids in the treatment of ocular inflammatory diseases, although additional safety evaluation should be undertaken. More detailed experiments are needed to evaluate the safety and efficacy of topical 8-oxo-dG for clinical applications (especially at various therapeutic concentrations not included in this study). We observed that the long-term use of 8-oxo-dG did not elevate intraocular pressure in a murine model compared to the use of corticosteroid. (unpublished data) Additionally, we plan to further investigate the effects of 8-oxo-dG in dry eye disease, allergic conjunctivitis, uveitis, and macular edema in subsequent studies.

In conclusion, our findings revealed that topical 8-oxo-dG demonstrated excellent therapeutic effects by suppressing inflammation and were comparable with those of a corticosteroid in an experimental ocular alkali model. We believe that topical 8-oxo-dG eye drops may be a promising, novel therapeutic strategy for various ocular surface inflammatory diseases including those caused by chemical injuries.

## Supplementary Information

Supplementary Information.
